# Undertaking a Collaborative Rapid Realist Review to Investigate What Works in the Successful Implementation of a Frail Older Person’s Pathway

**DOI:** 10.3390/ijerph15020199

**Published:** 2018-01-25

**Authors:** Éidín Ní Shé, Fiona Keogan, Eilish McAuliffe, Diarmuid O’Shea, Mary McCarthy, Rosa McNamara, Marie Therese Cooney

**Affiliations:** 1 School of Nursing, Midwifery and Health Systems, University College Dublin, Belfield, Dublin 4, Ireland; eilish.mcauliffe@ucd.ie; 2 The Ireland East Hospital Group, Mater Misericordiae University Hospital, Eccles Street, Dublin 7, Ireland; Fkeogan@iehg.ie; 3 St. Vincent’s University Hospital, Elm Park, 196 Merrion Rd., Dublin 4, Ireland; D.OShea@st-vincents.ie (D.O.); mariecooney@st-vincents.ie (M.T.C.); 4 Older Person’s Empowerment Network and Patient and Public Involvement Representative in Healthcare at the Health Service Executive, Dublin 8, Ireland; mmcarthy47@gmail.com; 5 St. James Hospital, James’s St., Dublin 8, Ireland; rosamcnamara@gmail.com; 6 School of Medicine, University College Dublin, Belfield, Dublin 4, Ireland

**Keywords:** frailty, older people, pathways, acute, realist review, co-design, implementation, stakeholder engagement

## Abstract

We addressed the research question “what factors enable the successful development and implementation of a frail older person’s pathway within the acute setting”. A rapid realist review (RRR) was conducted by adopting the RAMESES standards. We began with a sample of 232 articles via database searches supplemented with 94 additional records including inputs from a twitter chat and a hospital site visit. Our final sample consisted of 18 documents. Following review and consensus by an expert panel we identified a conceptual model of context-mechanism-(resources)-outcomes. There was overall agreement frailty should be identified at the front door of the acute hospital. Significant challenges identified related to organisational boundaries both within the acute setting and externally, the need to shift outcomes to patient orientated ones, to support staff to sustain the pathway by providing ongoing education and by providing role clarity. RRRs can support research such as the systematic approach to improving care for frail older adults (SAFE) study by producing accounts of what works based on a wide range of sources and innovative engagement with stakeholders. It is evident from our provisional model that numerous factors need to combine and interact to enable and sustain a successful frail older person’s pathway.

## 1. Introduction

Frail older people are vulnerable due to their loss of physiologic reserve across multiple organ systems, and can experience disproportionate loss of functional ability even when hospitalised for a relatively minor illness [1,2,3]. Delays to the clinical decision-making process have been found to prolong hospital stays exacerbating the problem further [4,5]. The literature suggests the best approach to prevent functional loss amongst older adults is to provide a comprehensive multidisciplinary integrated response [6]. For the majority of older persons in Ireland, the emergency department (ED) is the ‘front door’ of entry to acute care. A recent Department of Health special delivery unit report demonstrated that people aged over 65 year’s account for over one-third of ED attendances [7]. As older people often have complex requirements, the Irish healthcare system needs to adapt to meet these requirements, especially since the demand will increase as the population ages [8,9,10]. Recent attention has focused on identifying the best pathways for treating frail older patients. Previous studies illustrate the importance of continuing organizational support, clinical champions who communicate regularly with decision makers, dedicated staffing, and ongoing data collection [7,11].

One approach to improve pathways of care of frail older people is via the establishment of an acute frailty unit providing a multidisciplinary approach to care. Several units in the United Kingdom have assessed the benefits of acute frailty units (AFUs) demonstrated reduced admissions, shorter length of stay, avoidance of admission and increased percentages of admission <24 h [12]. Whilst the evidence that is emerging is positive very little information is available regarding the implementation of these strategies within the Irish context. We aimed to respond to this by undertaking a rapid realist review (RRR) of the literature as part of the Systematic Approach to Improving Care for Frail Older Patients (SAFE) study [13]. The aim of SAFE is to develop and explore the process of implementing a model of excellence in the delivery of patient-centred integrated care within the context of acute presentations of frail older people [13]. The project is funded by the Irish Health Research Board applied partnership award (APA). The grant specifications influenced the design of the study where the research must be applied by the knowledge user organizations [14]. The award supports projects where academic researchers and knowledge users must come collaborate to focus on themes/questions which are determined by the documented needs of the Irish health and social care system.

## 2. Methods

**Figure 1:**
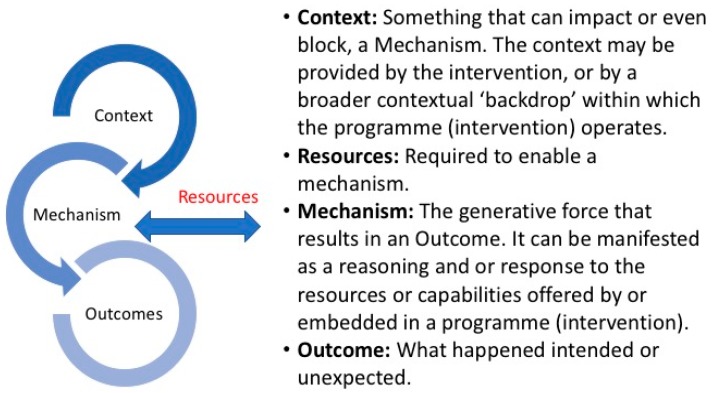
Rapid Realist Review of the Evidence—What works, for who and in what circumstances?

A RRR is focused on iteratively building on the empirical and theoretical literature by accommodating and summarizing a diversity of evidence types [15]. The foundations of the approach recognises that all interventions and programmes have underlying theories which can be either implicit or explicit that can impact these programs [16]. It is a flexible, theory driven approach that recognises the complexity of intervention in ‘the real world’. For those working within an applied clinical setting, an RRR is an appealing approach to enable the unpacking of the complexities of contexts and interrelated mechanisms underlying implementation activities [15,16]. Key to this is to enable the active participation and engagement of those working and using the intervention or programme. The benefits of engaging diverse stakeholders in the co-production of the literature review process is noted as being beneficial as it provides increased clarity and awareness of the transferability of the review findings [17]. Whilst a full realist review engages in a much longer exploration of the literature and a period of ‘testing’ an RRR, in contrast, have emerged to assist a speedier transition from research to practice [18]. They are used during the initial phase of a multiphase project where the research findings need to be rapidly adapted to for the next stage and where there are limited time and resources available. This suited the SAFE study as it is funded for a two year period. The basic question of an RRR is ‘what is it about this intervention that works in this context and why’? [18,19]. An RRR works on understanding what are the context’s (C), mechanisms (M), and outcomes (O) that enable or constrain the implementation of an intervention (Figure 1).

This RRR adheres to the RAMESES realist publication standards guide [20] however it has been adapted to streamline and accelerate the process as advised in the literature [18] via the establishment of an expert panel and reference panel. The process of both are outlined below.

### 2.1. Establishment of an Expert Panel

The expert panel comprises content experts who ensure the review reflects current thinking and are central to the success of an RRR [18,19]. They are tasked with providing relevant documents for review. This includes grey or operational documents, thus improving access to material not yet published. Secondly, the panel validates via consensus the final conceptual model. Panel involvement does not replace a literature search rather it provides a way of quickly identifying relevant material for tailoring a search strategy. Panelists draw on their expertise to ensure any interpretations of the literature fit with the experiences of those involved in the content area, as well as with the needs and realities of the local context. The expert panel convened in January 2017 consisting of seven members with experience in health systems, geriatric medicine, allied health, integrated care, co-design, emergency care and a member of the public with experience in public and patient involvement. Two members of the panel were not directly involved with the SAFE project but were identified as having relevant experience in implementing and working within frailty pathways both nationally and internationally (Keogan and McNamara Appendix A).

Following discussions amongst the expert panel on how to ensure that local knowledge would be captured it was agreed that reference panel would be engaged in two ways as presented below.

### 2.2. Establishment of Reference Panels

Reference panels are essentially sounding boards providing local contextual knowledge to ensure that the review and final conceptual model is inclusive to the experience of those ‘on the ground’ [17]. To capture the experiences from those working and accessing the health care system in Ireland the expert panel proposed two initiatives. The first was via the hosting of a twitter driven event via the curated #IrishMed chat. This is a discussion that takes place every Wednesday for an hour at 10 pm Irish time on all things relating to health. On average, between 180 and 200 participants take part sending between 1500–2000 tweets. Weekly hosts facilitate the discussion by providing four to five questions to direct the conversation in areas such as eHealth, Integrated Care and pain management. For the second reference panel, it was agreed to undertake a site visit and a workshop with the multidisciplinary frail intervention therapy team (FITT) based in Beaumont hospital in Dublin. The FITT team is dedicated to identifying frailty in the ED and providing an early comprehensive multidisciplinary assessment to patients. Both methods of engagement it was agreed would be synthesized to contribute to the RRR conceptual model.

### 2.3. Research Questions and Search Strategy

The scope of the RRR was agreed by the expert panel at the first inception meeting in January 2017. The overarching RRR question finalised was to understand “what factors enable the successful development and implementation of a frail older person’s pathway within the acute setting”. The primary and secondary exclusion criteria and search terms for the academic search was agreed by the expert panel (Appendix B). Following consultation with a university faculty librarian, two members of the team Ní Shé and Cooney undertook a search of the literature using Cochrane, PubMed, and CINAHL databases (Appendix C). This search was supplemented with key articles and other documents relevant from the grey literature as identified by the expert and reference panels.

Four questions were designed by the expert panel for the co-hosted twitter chat that occurred on the 11 January 2017 (Appendix D). A total of 164 participants took that part in the hour long facilitated discussion sending 1263 tweets within participants contributing from the USA, Canada, United Kingdom and Australia [20]. Data was extracted and summarised that was included in the final conceptual model.

The workshop with the FITT team was held in Beaumont hospital on the 9 February 2017 which was attended by three members of the team Cooney, Keoghan and Ní Shé to capture what had worked for them in developing and sustaining the FITT model [21]. A summary of the workshop was synopsised and incorporated into the RRR.

## 3. Data Extraction and Analysis

Data was extracted from January to April 2017 with fortnightly meetings to critically appraise, analyse and synthesise the data using a data extraction tool (Appendix E). Data extraction included:

1. Background information such as the setting and demographics to outline possible contextual factors;
2. Key workings that contributed to the design and functioning of a pathway to identify mechanisms and resources;
3. Information and evidence suggestive of the successes or failures of different aspects of an intervention.

We specifically attempted to identify context, mechanisms, resources and outcomes that had been outlined to enable the sustainability of a frailty pathway within the acute setting. All extractions were reviewed by the synthesis lead (ÉNS) for possible C-M(R)-O. Resources (R) was included to capture what is required to enable mechanisms. A final consensus meeting was held by the expert panel in June 2017 to agree on a C-M(R)-O for operationalising in the next stage of the SAFE study [13].

## 4. Results

### 4.1. Nature of Dataset

**Figure 2:**
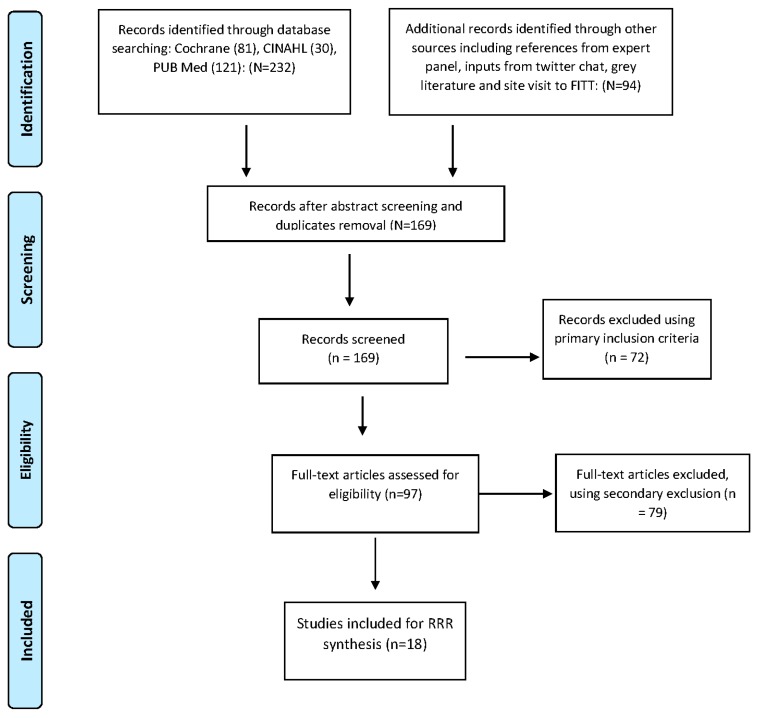
Modified PRISMA flow diagram of data search.

Our final review consisted of 18 documents (see Figure 2) consisting of the following:

- Five systematic/scoping reviews: This included a review of the effectiveness of ED and community transitions [6], review of interventions of comprehensive geriatric assessments in the ED [7] scoping of the structure and process for geriatric multidisciplinary teams [22], the feasibility of identifying frailty in the ED [3] and a systematic review on the feasibility and implementation of frailty identification tools within the emergency department [23].
- Three grey literature articles/reports: Including the Beaumont hospitals FITT program operational model [21], a UK report on older people’s contribution and understanding and preventing avoidable hospital admissions [24], and the UK report on hospital care of frail older people [25].
- Two editorials/summary pieces: An overview of new horizons of urgent care of frail older people [2] and on what works within emergency care for frail older people [26].
- Six studies/interventions within one acute site: UK study on older patient’s flow through hospital to improve efficiency and reduce mortality [27]; an evaluation of an ED frailty unit in the UK [28], geriatric ED intervention in Australia [29], outcome study of frailty indicators in Sweden [30], US study on the impact of an eat walk engage program [31] and on embedding a comprehensive geriatric assessment unit [32].
- Two multi-site studies/interventions: A Dutch study of implementing comprehensive geriatric care programs to reduce functional decline of older patients in three hospital sites [1], and a UK study of care transitions of frail older people within six sites [33].

**Figure 3:**
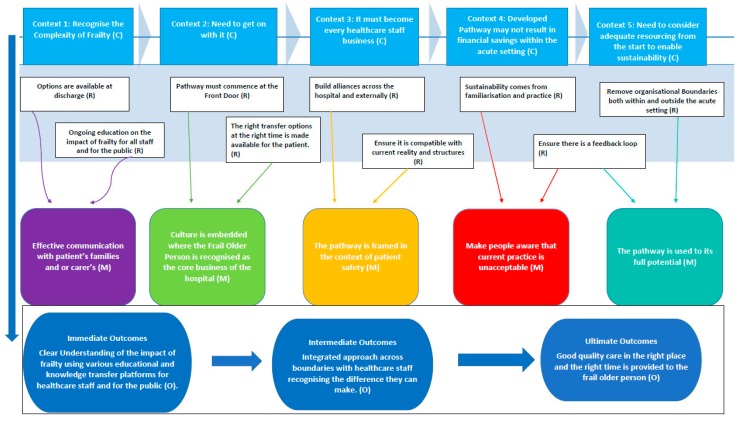
Conceptual Model: What factors enables the successful development and implementation of a frail older person’s pathway within the acute setting?

The expert panel via a consensus meeting validated and prioritized the conceptual model (Figure 3). We identified from the review and the final consensus meeting five contexts, five mechanisms, nine linked resources and three outcomes that enable the successful implementation (Figure 3). Below provides a summary overview of the main themes that emerged from the synthesis resulting in the conceptual model.

### 4.2. Identify Frailty at the Front Door—Emergency Department

Frailty is associated with key clinical syndromes including loss of mobility, falls, confusion, incontinence and polypharmacy [13]. Frail patients are vulnerable to adverse effects of hospitalisation including deconditioning, immobility, and loss of independence [22]. There is an overall agreement that acute hospitals should identify frailty at the earliest possible opportunity considered to be at the ‘front door’ emergency department [4,6,7,21,22,23,24,25,26,33]. However, there is a lack of good recent quality evidence to guide the care of frail older people attending the emergency department [4]. One systematic review undertaken in 2015 focused on trials of transitional care models supporting the discharge of older people in six countries from EDs found that all the trials were at least five years old and noted that there was limited high-quality data available [6]. The review noted that very few studies utilised research evaluation design and concluded that the primary outcome utilised was an early return to the ED or hospital admission as measurements of effectiveness. The follow-up periods ranged from fourteen days to eighteen months. The authors suggest that complexity of older people health needs to be recognised and advocates that more patient-family and carer-centred outcomes be prioritised such as independence in assisted daily living, patient experience and family and carer pressure [6]. A focus on patient centred outcomes rather than on reduction of bed days is stressed as what should be a key priority [4,29]. A recent systematic review [23] on the feasibility of identifying frailty in the ED found nine tools which were quick to us that took less than ten minutes to use however only 50% of eligible patients were screened. The review found a lack of information available in the literature on the practical details of implementing or administrating the tools in the acute setting. The paper suggests that further work was required to clarify who would use the tool and when to clarify they are acceptable in practice.

### 4.3. Remove Organisational Boundaries and Be Clear on Workloads

Frail older people experience frequent care transitions [27]. A significant challenge noted in the literature related to organisational boundaries both within the acute setting and externally with the community and or with rehabilitation partners [1,22,28,30,31,33]. Often staff had few opportunities to build trust or understand each other’s service provision capacities [21]. Suggestion’s to assist included the establishment of forums for staff to build relationships and to support staff to work in a more integrated way and to provide on-going education [21,22,33]. One specific paper from Australia [31] reinforced the need to have local champions and provide a holistic enabling approach to address local contextual barriers. Clarity on participant’s workloads and having adequate resource to enable sustainability was also stressed. The Australian study observed that often less skilled tasks such as assisting with meals or providing hydration were less likely to have champions and a significant challenge to sustainability and a significant barrier with exiting staffing structures [29]. A multi-site Dutch study found a number of organisational factors in implementing a comprehensive geriatric program [1]. The authors note that even with scientifically sound program sustained engagement across all levels of an organisation is required to ensure sustainability.

### 4.4. Set Realistic Time Frames, Support Staff and Make It Everyone’s Business

All staff across and outside the system need to be educated on frailty and on the specific needs of frail older people who can often have complex needs [1,23,24,30,32]. Geriatricians and their teams can act as key enablers building alliances to support inter-professional collaboration and to enable gradual and sustained improvements [4,31]. Significantly the literature recognised that the scale and complexity of implementing and sustaining an integrated approach can easily overwhelm with even strong leadership. Key to the success is adapting the activities to the local context whilst recognising that changes to practice will take longer to achieve than anticipated [23,26]. When rolling out a pathway consideration and support should be provided to staff who may not be familiar with working the ED. This was noted by some members the multidisciplinary FITT team [21] who had not previously worked in the ED and found the initial experience challenging.

### 4.5. Engage with Older People and the Developed Pathway May Not Result in Financial Savings

Two grey literature reports were significant in highlighting that even when there was better integration between health and social care that it may not result in financial saving for the acute sector [24,25]. Both reports stressed that community care often only postpones the need for hospital care of frail older people. Significantly there is a need to recognise that older people are experts by their experiences and they and their carers must be included and consulted [24]. Key to this is to utilise methodologies and approaches that empower and includes their perspectives [24,25].

## 5. Discussion

This to the best of our knowledge, is the first RRR reviewing the factors the enable the successful development and implementation of a frail older person’s pathway within the acute setting. In contrast to a systematic review, the RRR builds an understanding of why and how things work. Therefore, the literature presented in this paper points to a variety of factors that we need to consider as we advance the SAFE study. We acknowledge that whilst an RRR is not a comprehensive search, the methodology allowed us to proceed with a much broader engagement with the public and with those working the health system both nationally and internationally via the expert and reference panel process. There were certainly gaps within the literature with limited available for example to perform an economic analysis or evidence capturing long-term sustainability. This RRR process has drawn from diverse literature both grey and academic supplemented with the insights from the reference panel process and final consensus by the expert panel. The review results are not surprising and points specifically for the need to shift outcomes to more patient orientated ones (24, 25, 30). Early co-design work with our public and patient partners on the SAFE study have prioritised several outcomes. These will be tested in the next stage of the SAFE study using plan-do-study-act cycles. We have also undertaken qualitative work within health workers within the acute, community and rehab settings that confirms many of the barriers identified in this review. In particular the work highlights a lack of ongoing education and a lack of communication between organisations. Also an earlier attempt to embed a pathway had failed due to a lack of resources, champions or ongoing education to sustain it. Clarity is provided in the literature that frailty should be identified at the ‘front door’ of the hospital the emergency department. There are a number of tools available for measurement. However, further research is required to capture acceptability and understanding as to who will undertake the frailty screening.

The significant strength of this study was the RRR process enabled us to capture local contextual factors via our two way reference panel process. The insights shared by the twitter participants and the FITT team in establishing their frailty pathway captured valuable insights that were not available in the literature and enhanced the richness of the review. The expert panel via consensus established a conceptual model to enable understanding of the complex processes surrounding the implementation of a frail older person’s pathway within the acute setting (Figure 3). This framework is shaping the next stage of the SAFE study.

## 6. Conclusions

RRRs can support a multiphase project such as the SAFE study by producing accounts of what works based on a wide range of sources and engaging with diverse stakeholders. There is now extensive and robust evidence in the literature that frailty should be identified in the ED and comprehensive evidence of the types of measurement tools available. It is evident from our conceptual model that numerous factors need to combine and interact to enable and sustain a successful frail older person’s pathway. These include clarity of workloads, removing organisational barriers, supporting staff through ongoing education, including public and patient perspectives in identifying outcomes. A significant finding in this study was the absence in the academic literature including patients and the public groups in identifying outcomes. The SAFE study will respond to this gap by co-designing the pathway with diverse partners to enable the development and sustainability of a pathway.

## Abbreviations

RRR	rapid realist review
ED	Emergency Department
SAFE	systematic approach to improving care for frail older adults

## Appendix A: Expert Panel Membership

- Dr. Marie Therese Cooney, Clinical Lecturer & Consultant Geriatric Medicine and Acute Medicine, St Vincent’s University Hospital;
- Dr. Diarmuid O’Shea, National Clinical Lead for Programme for Older Persons and Consultant Geriatric Medicine and head of department medicine for the elderly, St Vincent’s University Hospital;
- Ms. Mary McCarthy, Member of Patient and Public Involvement in Healthcare at the HSE and Co-Chair of the SAFE Public and Patient co-design panel;
- Ms. Fiona Keogan, Frailty Lead, Ireland East Hospital Group formally Head of Clinical Services & Business Planning, Beaumont Hospital, Dublin;
- Dr. Rosa McNamara, Consultant in Emergency Medicine at St James’s Hospital Dublin formally Imperial College Healthcare NHS Trust);
- Professor. Eilish McAuliffe, Professor of Health Systems, School of Nursing Midwifery and Health Systems, University College Dublin;
- Dr. Éidín Ní Shé, Health Systems researcher, School of Nursing Midwifery and Health Systems, University College Dublin.

## Appendix B: Inclusion and Exclusion Criteria and Database Search Terms

The primary and secondary exclusion criteria and search terms for academic search:

**Primary Exclusion Criteria** Studies not written in English; Studies that include participants who are not human; Studies where the primary focus was not on the care of frail older people; Studies which were letters, notes, conference abstracts or reviews only;
**Secondary Exclusion Criteria** Studies where there was no description of any intervention or guidelines; Studies that did not report any outcome or results; Studies where there were no acute care elements; Unable to obtain further information to make assessment;
**Search Terms Used** (Frail older person pathway, or ‘frail elderly pathway’ or “frailty units”) and (intervention or interventions or strategy or quality improvement) and (acute setting)

Inclusion criteria descriptions:

1. Articles discusses what works to support implementation of frail older person pathways in the acute setting;
2. Articles discusses the implementation of acute frailty units in the acute setting;
3. Article discusses frailty within the context of older persons within the acute setting;
4. Articles discusses the health policies supporting the care of frail older persons within the acute setting and that offers recommendations for the future based on research evidence and stakeholder’s experiences including public and patient involvement;
5. Articles discusses potential barriers to the care of frail older persons within the acute setting;
6. Articles that discusses what resources enabled the development and implementation of a frail older person pathways to be successfully implemented within the acute setting;
7. Articles discusses how hospitals and the community setting are working together in implementing a frail older persons pathway;
8. Articles discussing frailty measurement.

## Appendix C: Search Terms

**Cochrane**	**Cinahl**	**Pub Med**
Search 1. “frail older person” AND “Pathway”	Search 1: “frail older person” AND “Pathway” AND “Intervention	Search 1: “Frail Elderly” AND “Pathway” AND “Intervention”
Search 2. “Acute Frailty Units”	Search 2 “Frail elderly person” AND “Pathway” AND “Intervention”	Search 2: “Acute frail elderly” AND “emergency department”
Search 3. “frail elderly” AND “Pathwa”	Search 3: “Frail elderly” AND “Intervention” AND “Emergency Department”	Search 3: “Frail elderly” AND “Quality Improvement” AND “Emergency Department”
	Search 4 “Frail elderly” AND “Quality Improvement” AND “Emergency Department”	
Total: 81 for screening	Total: 30 for screening	Total: 121 for screening

## Appendix D: Developing a Frail Older Person’s Pathway—SAFE #IrishMed Tweetchat 11/1/17

- T.1. Why is there a need to develop frail older person’s pathways within the acute setting?
- T.2. How should it look like in Ireland? What should be included in a pathway? What should we use to measure frailty?
- T.3. What role can the community play in frail older persons pathways? How can the community and the hospital better integrate?
- T.4. What evidence of best practice is available that you can recommend?

## Appendix E: Synthesising Tool Template

**Title**		**Year**	
Author(s)		Funders	
Key Words		Setting	
Research Questions		Study Location	
Sample Size		Population	
Primary Outcome		Secondary Outcome	
Intervention	Context	Mechanisms	Outcomes
Extras		Notes-Resources outlined	

## References

[1] De Vos A.J.B.M., Asmus-Szepesi K.J., Bakker T.J.E.M., de Vreede P.L., van Wijngaarden J.D., Steyerberg E.W., Mackenbach J.P., Nieboer A.P. (2012). Integrated approach to prevent functional decline in hospitalized elderly: The Prevention and Reactivation Care Program (PReCap). BMC Geriatr..

[2] Clegg A., Young J., Iliffe S., Rikkert M.O., Rockwood K. (2013). Frailty in elderly people. Lancet.

[3] Chong E., Ho E., Baldevarona-Llego J., Chan M., Wu L., Tay L. (2017). Frailty and Risk of Adverse Outcomes in Hospitalized Older Adults: A Comparison of Different Frailty Measures. J. Am. Med. Dir. Assoc..

[4] Conroy S., Turpin S. (2016). New horizons: Urgent care for older people with frailty. Age Ageing.

[5] Gill T.M., Gahbauer E.A., Allore H.G., Han L. (2006). Transitions between frailty states among community-living older persons. Arch. Intern. Med..

[6] Lowthian J., McGinnes R., Brand C., Barker A., Cameron P. (2015). Discharging older patients from the emergency department effectively: A systematic review and meta-analysis. Age Ageing.

[7] Ellis G., Whitehead M., O’Neill D., Langhorne P., Robinson D. (2011). Comprehensive geriatric assessment for older adults admitted to hospital. Cochrane Database Syst. Rev..

[8] McLoughlin J. (2014). The Case for a Radical Overhaul of the Care Pathways for the Elderly in the Emergency Department.

[9] Wren M., Keegan C., Walsh B., Bergin A., Eighan J., Brick A., Connolly S., Watson D., Banks J. (2017). Projections of Demand for Healthcare in Ireland, 2015–2030: First Report from the Hippocrates Model.

[10] Central Statistics Office (2013). Population and Labour Force Projections.

[11] Rubin F.H., Neal K., Fenlon K., Hassan S., Inouye S.K. (2011). Sustainability and scalability of the hospital elder life program at a community hospital. J. Am. Geriatr. Soc..

[12] Steelfisher G.K., Martin L.A., Dowal S.L., Inouye S.K. (2011). Sustaining clinical programs during difficult economic times: A case series from the hospital elder life program. J. Am. Geriatr. Soc..

[13] Cooney M.T., O’Shea D., Shé É., McCarthy M., O’Donnell D., McAuliffe E., Slater N., Collins O., Hughes G., Cogan L. (2017). Co-designing a Systematic Approach to improving care for Frail Older Patients. Int. J. Integr. Care.

[14] Health Research Board (2016). Applied Partnership Award (APA). http://www.hrb.ie/research-strategy-funding/grants-and-fellowships/hrb-grants-and-fellowships/grant/152/.

[15] Rycroft-Malone J., McCormack B., Hutchinson A.M., DeCorby K., Bucknall T.K., Kent B., Schultz A., Snelgrove-Clarke E., Stetler C.B., Titler M. (2012). Realist synthesis: Illustrating the method for implementation research. Implement. Sci..

[16] Khangura S., Polisena J., Clifford T.J., Farrah K., Kamel C. (2014). Rapid review: An emerging approach to evidence synthesis in health technology assessment. Int. J. Technol. Assess. Health Care.

[17] Lavis J.N., Røttingen J., Bosch-Capblanch X., Atun R., El-Jardali F., Gilson L., Lewin S., Oliver S., Ongolo-Zogo P., Haines A. (2012). Guidance for evidence-informed policies about health systems: Linking guidance development to policy development. PLoS Med..

[18] Saul J., Willis C., Bitz J., Best A. (2013). A time-responsive tool for informing policy making: Rapid realist review. Implement. Sci..

[19] Wong G., Greenhalgh T., Westhorp G., Buckingham J., Pawson R. (2013). RAMESES publication standards: Realist syntheses. BMC Med..

[20] Storify Developing a Frail Older Person’s Pathway—SAFE #IrishMed Tweetchat. https://storify.com/drlfarrell/irishmed-5876c60c40f3000e17c572d9.

[21] Beaumont Hospital (2017). Frailty Therapy Intervention Team-Summary Document and Summary Notes from Workshop with FITT Team in Beaumont Hospital.

[22] Deschodt M., Claes V., Van Grootven B., Van den Heede K., Flamaing J., Bolan B., Milisen K. (2016). Structure and processes of interdisciplinary geriatric consultation teams in acute care hospitals: A scoping review. Int. J. Nurs. Stud..

[23] Elliott A., Hull L., Conroy S. (2017). Frailty identification in the emergency department—A systematic review focussing on feasibility. Age Ageing.

[24] Glasby J., Littlechild R., Le Mesurier N., Thwaites R. (2016). Who Knows Best? Older People’s Contribution to Understanding and Preventing Avoidable Hospital Admissions.

[25] (2014). HSJ/Serco Commission on Hospital Care for Frail Older People: Main Report. https://www.hsj.co.uk/5076859.article.

[26] Conroy S. (2015). Emergency care for frail older people-urgent AND important-but what works?. Age Ageing.

[27] Silvester K., Mohammed M., Harriman P., Girolami A., Downes T. (2014). Timely care for frail older people referred to hospital improves efficiency and reduces mortality without the need for extra resources. Age Ageing.

[28] Conroy S., Ansari K., Williams M., Laithwaite E., Teasdale B., Dawson J., Mason S., Banerjee J. (2014). A controlled evaluation of a comprehensive geriatric assessment in the emergency department: The ‘Emergency Frailty Unit’. Age Ageing.

[29] Craswell A., Marsden E., Taylor A., Wallis M. (2016). Emergency Department presentation of frail older people and interventions for management: Geriatric Emergency Department Intervention. Saf. Health.

[30] Eklund K., Wilhelmson K., Gustafsson H., Landahl S., Dahlin-Ivanoff S. (2013). One-year outcome of frailty indicators and activities of daily living following the randomised controlled trial; “Continuum of care for frail older people”. BMC Geriatr..

[31] Mudge A., McRae P., Cruickshank M. (2015). Eat Walk Engage: An Interdisciplinary Collaborative Model to Improve Care of Hospitalized Elders. Am. J. Med. Qual..

[32] Taylor J., Gaillemin O., Pearl A., Murphy S., Fox J. (2016). Embedding comprehensive geriatric assessment in the emergency assessment unit: The impact of the COPE zone. Clin. Med..

[33] Baillie L., Gallini A., Corser R., Elworthy G., Scotcher A., Barrand A. (2014). Care transitions for frail, older people from acute hospital wards within an integrated healthcare system in England: A qualitative case study. Int. J. Integr. Care.

